# High-efficient white blood cell separation from whole blood using cascaded inertial microfluidics

**DOI:** 10.1016/j.talanta.2024.127200

**Published:** 2024-11-14

**Authors:** Haotian Cha, Xiaoyue Kang, Dan Yuan, Belinda de Villiers, Johnson Mak, Nam-Trung Nguyen, Jun Zhang

**Affiliations:** a Queensland Micro- and Nanotechnology Centre, Griffith University, Nathan, Queensland, 4111, Australia; b School of Mechanical and Mining Engineering, The University of Queensland, Brisbane, QLD, 4072, Australia; c Institute for Glycomics, Griffith University, Gold Coast, Queensland, Australia; d School of Engineering and Built Environment, Griffith University, Nathan, Queensland, 4111, Australia

## Abstract

White blood cells (WBCs) are a crucial component of the human immune system. WBCs contain invaluable information about the health status of the human body. Therefore, separating WBCs is indispensable for the diagnosis of many diseases in clinical setting. The low ratio of WBCs to red blood cells in whole blood has made the isolation of WBCs challenging. As the conventional single-stage microfluidic technology cannot provide sufficient separation purity. We used a cascaded inertial microfluidic chip by consecutively connecting two sinusoidal channels to enhance the purity of WBCs after single processing. The improvement was in part due to the diversion of the sample at the end of the first stage separation, resulting in a lower flow rate in the second stage of processing within the cascaded device. We embedded concave micro-obstacles in sinusoidal channels to adjust their effective working flow rate range and enable the proper operation of both channels simultaneously. Using polystyrene beads mixture (5 and 10 μm) with a primary ratio of 1000 to 1, a single processing step through our cascaded chip improved the purity of 10-μm particles with more than three orders of magnitude of enrichment (from 0.08 % to 99.83 %) with a flow rate of 560 μL/min (Re = 77). Using diluted whole blood (× 1/10), we achieved 307-fold enrichment of WBCs (0.14 %–43.017 %) in a single process which was accompanied with ~3 orders of magnitude background removal of RBCs (from 4.8 × 10^8^ to 5.7 × 10^5^ counts/mL). This cascaded manner chip has the capacity to achieve high-efficiency separation of blood cells for clinical diagnosis.

## 1. Introduction

Human blood contains a multitude of information that reflects physiological conditions, consisting of blood plasma and blood cells. Blood cells primarily comprise platelets, red blood cells (RBCs), and white blood cells (WBCs) [1,2]. Platelets are the smallest blood cells with an anuclear shape, a diameter ranging from 2 to 4 μm, and a concentration of (2–5) × 10^8^ counts/mL. RBCs are anucleate cells with a discoid shape, a diameter of 6–8 μm and a thickness of ~2 μm. RBCs are the most abundant blood cells, accounting for about 40–45 % of to total blood volume. The concentration of RBCs is approximately (4–6) × 10^9^ counts/mL. WBCs are nucleated and generally spherical, with 8–12 μm in diameter, constituting around 1 % of total blood volume with a concentration of (5–10) × 10^6^ counts/ml [3–6]. In clinical diagnostics, blood tests serve as pivotal indicators [7]. For instance, RBCs are critical in assessing the severity of conditions such as sickle cell anaemia, blood disorders, and end-stage renal disease [8,9]. WBCs, integral components of the human immune system, play an indispensable role in diagnosing various illnesses, including Acquired immunodeficiency syndrome (AIDS), autoimmune diseases, immune deficiencies, leukaemia, appendicitis, and more [10–12]. Therefore, isolating WBCs from a massive cell environment with a high purity is essential for WBCs-related analysis or diagnosis. However, WBCs separation from whole blood is challenging because of their low (≤1 %) concentration. The conventional methods for blood separation in the laboratory are centrifugation and filtration. Centrifugation, the most common method, relies on the principle of differential gravity created by the centrifuge’s rotation to achieve separation. However, it is time-consuming and labour-intensive [13,14]. Filtration employs a membrane-type plasma separator to separate blood cells. However, membrane blockage can occur, and high flow rates can cause cell rupture. These two methods are relatively comparable in term of efficiency [15,16].

Microfluidic technologies can manipulate and process a small amount of liquid sample in micro- or nanoscale devices [17–19]. This method offers high sensitivity, cost-effectiveness, and rapid analysis capabilities, making it well-suited for processing blood cells while ensuring precise and timely analytical outcomes [20,21]. To isolate specific cells from the whole blood, many microfluidic methods have been developed based on active separation through the use of electric [22], acoustic [23,24], and magnetic [25] forces or passive separation achieved with channel geometry [26,27] or viscoelastic fluids [28,29] Among them, inertial microfluidic technology has attracted attention due to its advantages of structure simplicity, label-free, high-throughput and precise manipulation [30–34]. At high flow speeds within an curved microchannel, inertial lift forces and secondary flows are induced [35]. Dispersed particles migrate to the specific equilibrium positions inside a microchannel, known as inertial migration, by the counterbalance of the inertial lift force and secondary flow drag force [36]. Since the equilibrium locations and numbers of particle migration depend on the size of particles, it could result in the separation of particles with different sizes.

Inertial microfluidics can be a powerful tool for WBC separation. Spiral inertial microfluidics have been widely investigated. Zhu et al. developed an innovative inertial microfluidic cube featuring an embedded spiral-type microchannel. The system was integrated with lysis, storage and extraction modules to harvest WBCs from the blood sample [37]. However, the system requires an additional of hypotonic red blood cell lysis buffer, adding complexity and necessitating a higher degree of coordination between modules, which diminishes the desired user-friendliness. Jeon et al. developed a device based on a multi-dimensional double spiral (MDDS) device to isolate WBCs from a diluted blood sample [38]. Although four MDDS devices were integrated to improve the processing throughput, the initial sample with × 500 dilutions significantly reduce the throughput capacity. Mehran et al. proposed a spiral microfluidic channel with a U-shape cross-section to separate WBCs from the diluted blood (× 100). Still, this approach was limited in the throughput and high dilution times [39]. Regarding serpentine channel structure, our group introduced a continuous and high-throughput microfluidic platform for WBC separation, achieving a high flow rate of 288 mL/h for a diluted (× 1/20) whole blood sample by employing parallelisation designs. The recirculated processes by re-loading the outlet collection from the first process can achieve a high purification of WBCs with 48 % and around ten times enrichment ratio [3]. However, manual outlet collection and reloading between two processing stages are troublesome and labour-intensive. Therefore, a device with integrated multi-stage processing could offer greater potential, enabling sample processing and purification in a single run.

**Fig. 1.**
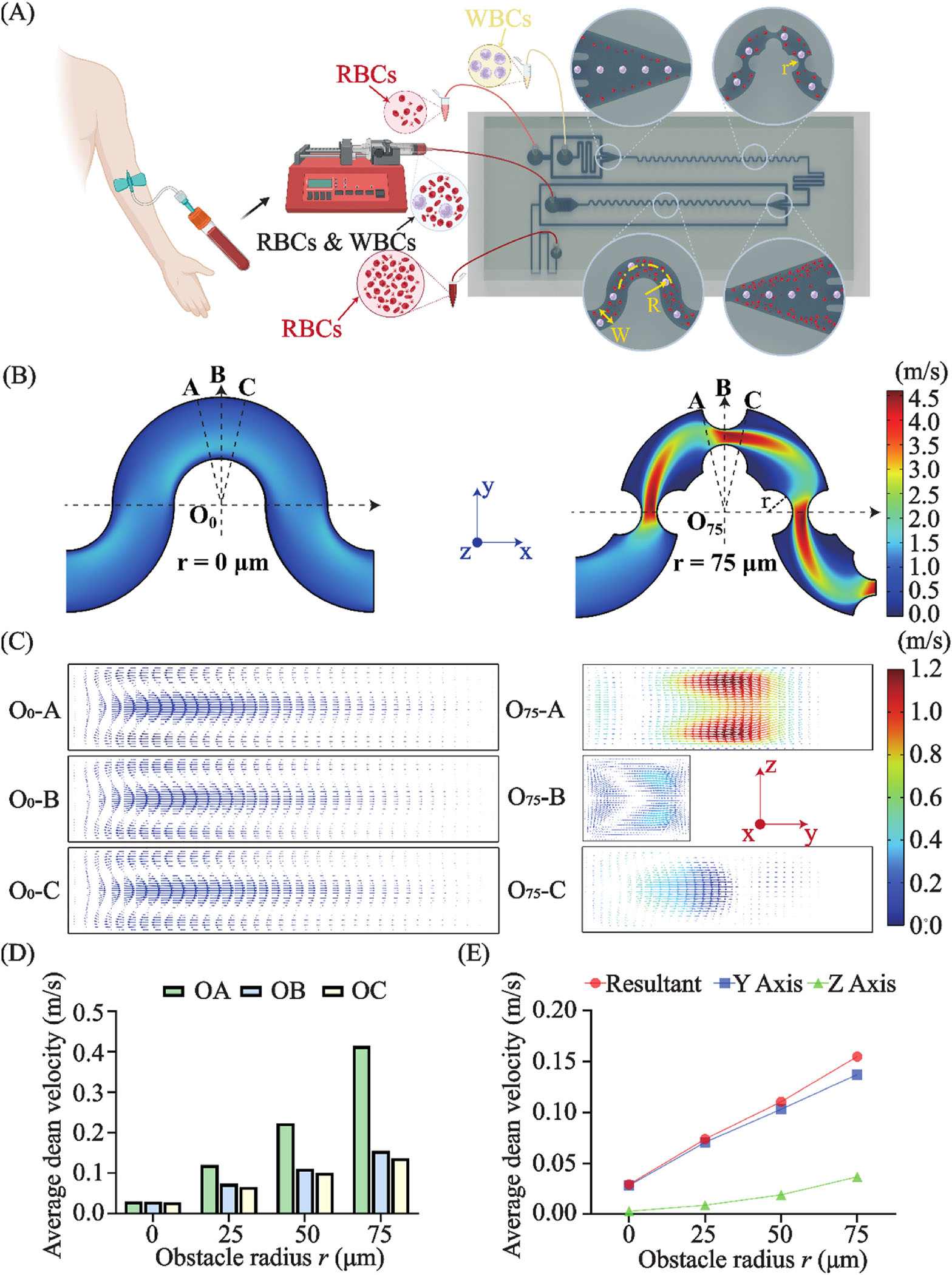
(A) Schematic diagram of separation of white blood cells (WBCs) from whole blood in the cascaded serpentine channels. (B) Numerical simulation of the main flow in the plain sinusoidal channel (without concave obstacles) and concave obstacle channel and (C) Dean flow at different cross-sections. The colour legend indicates the velocity magnitudes of the main flow and Dean flows. The arrows represent the Dean flow velocity within the cross-section. (D) The average magnitude of Dean velocity at cross-sections O-A, O–B and O–C of sinusoidal channels with different concave obstacle sizes. (E) The magnitude of Dean flow and its velocity components along Y and Z axes at cross-section O–B under different concave obstacle sizes. The flow rate is 350 μL/min (*Re* = 48).

In this work, we tested a cascaded inertial microfluidic device integrating two consecutive sinusoidal microchannels to separate WBCs from the blood sample, Fig. 1(A). We first investigated the effect of micro-obstacles on particle focusing, finding that micro-obstacles can be used to adjust the flow rate for optimal particle inertial focusing and separation. This insight guided the development of a cascaded device with two sequential sinusoidal microchannels, where the second stage incorporates micro-obstacles to fit the flow rate range and enable simultaneous separation. Testing with a binary particle mixture (5 and 10 μm, ratio ~1000:1), the device improved the purity of 10-μm particles from 0.08 % to 99.83 %, achieving a 1247-fold enrichment at a flow rate of 560 μL/min (Re = 77). Using diluted (× 1/10) whole blood, WBC purity increased from 0.14 % to 43.02 %, with a 307-fold enrichment in a single processing. We envisage that device with cascaded channels has the potential for high-efficiency separation of blood cells for clinical diagnosis and therapeutics.

## 2. Materials and methods

### 2.1. Design and fabrication

The microchannel is comprised of 20 replicated sinusoidal sections. The channel width (*W*) and height (*H*) are 200 and 40 μm, respectively. The curvature radius (*R*) is 250 μm. The semi-circular micro-obstacles are incorporated on the two sidewalls to form periodic local constrictions. Three different micro-obstacle radii (*r* = 25, 50 and 75 μm) were designed to investigate the effects of obstacle size on particle inertial focusing and separation. Besides, we designed a microfluidic device where two sinusoidal channels are connected in series so that the sample can undergo dual processing through the cascaded channels in a single run. The channel width (*W*), height (*H*), and curvature radius (*R*) for the two cascaded sinusoidal channels are identical, and the only difference is the size of the micro-obstacles. The micro-obstacle radii (*r*) in the cascaded sinusoidal channels are 0 and 75 μm, respectively, Fig. 1(A). All the polydimethylsiloxane (PDMS) microfluidic devices were fabricated using standard photolithography and soft lithography techniques [40].

### 2.2. Particles sample preparation

Spherical polystyrene microbeads of 5-μm (Thermo Fisher Scientific, product no. G0500) and 10-μm (Thermo Fisher Scientific, product no. G1000) diameters were used as the surrogates of red blood cells (RBCs) and white blood cells (WBCs). These microbeads were dispersed within deionised (DI) water with 0.1 % Tween 20 (Sigma-Aldrich, product no. P9416) to avoid particle aggregation. The particle concentrations are 7.19 × 10^5^ counts/mL and 8.99 × 10^4^ counts/mL for 5-μm and 10-μm particles, respectively. To characterise the device performance on particle separation, a mixture of 5-μm (Thermo Fisher Scientific, product no. G0500) and 10-μm (Phosphorex, product no. 010 KR) particles was prepared in a similar ratio (1000:1) of red blood cells and white blood cells in the whole blood. Particle concentrations of the 5-μm and 10-μm microbeads were approximately 2.72 × 10^8^ counts/mL and 2.72 × 10^5^ counts/mL, respectively.

### 2.3. Blood sample preparation

Blood was obtained from the Australian Red Cross Blood Service, and Griffith University Human Research Ethics approved the use of human blood samples under protocol number 2021/598. All experiments were conducted following the relevant laws and institutional guidelines. The concentration of RBCs and WBCs, measured by a full blood count, are 4.8 × 10^9^ and 6.7 × 10^6^ counts/mL, respectively, in whole blood samples. The whole blood was diluted 10 times using Hank’s Balanced Salt Solution (HBSS, 14025092, Gibco). We used DAPI (D9542, Sigma-Aldrich) to stain the nuclei of WBCs to distinguish WBCs from RBCs since RBCs have no nuclei. To prepare the DAPI solution, we first dissolved 1 mg DAPI into 1 mL Milli Q water, achieving a stock solution of 1-mg/mL concentration. Subsequently, we diluted the stock solution in HBSS and obtained a concentration of working DAPI solution of 1.5 μg/mL. Next, we added working DAPI solution into the samples of inlet and outlets with a final concentration of DAPI as 1 μg/mL. We placed aluminium foil over the sample and incubated it at room temperature for 45 min. Finally, we quantified the concentration of WBCs and RBCs using a haemocytometer (718620, BLAUBRAND Neubauer Pattern, BRAND, Germany).

### 2.4. Experimental setup and data analysis

The microfluidic devices were positioned on an inverted microscope stage (IX73P1F, OLYMPUS, Japan). A syringe pump (ISPLab02, DK infusetek, China) was utilised for the controlled infusion of particle samples into the devices. The infusion flow rate varied from 50 μL/min to 650 μL/min, with 50 μL/min intervals. A high-speed camera system (VEO, Photron, USA) was affixed to the microscope to record particle positions and fluorescence trajectories in the microchannels. The open-source software ImageJ (National Institute of Health) was employed to process and analyse videos and images.

Three pivotal criteria - purity, recovery, and enrichment ratio - were used to evaluate separation performance. Purity is defined as the ratio of target particles number to the total number of particles at the same outlet/inlet [41].


(1) $$\mathrm{Purity}=\frac{{\left[N_{\mathrm{target}}\right]}_{\mathrm{outlet}/\mathrm{inlet}\;}}{{\left[N_{\mathrm{total}\;}\right]}_{\mathrm{outlet}/\mathrm{inlet}\;}}$$

Recovery, or separation efficacy, is characterised by the ratio of target particles/cells at the specified outlet to the total counts of target particles/cells at the inlet [42].


(2) $$\mathrm{Recovery}=\frac{{\left[N_{\mathrm{target}}\right]}_{\mathrm{outlet}}}{{\left[N_{\mathrm{target}}\right]}_{\mathrm{inlet}}}≅\frac{{\left[N_{\mathrm{target}}\right]}_{\mathrm{outlet}}}{\sum_{\mathrm{all}}{\left[N_{\mathrm{target}}\right]}_{\mathrm{outlets}}}$$

Meantime, the enrichment ratio is defined as the ratio of particle/cell purity at the outlet ($P_{\mathrm{outlet}}$) to that at the inlet ($P_{\mathrm{inlet}}$) [43]:

(3) $$\mathrm{Enrichment}=\frac{P_{\mathrm{outlet}}}{P_{\mathrm{inlet}}}=\frac{{\left[\frac{N_{\mathrm{target}}}{N_{\mathrm{total}}}\right]}_{\mathrm{outlet}}}{{\left[\frac{N_{\mathrm{target}}}{N_{\mathrm{total}}}\right]}_{\mathrm{inlet}}}$$

## 3. Results and discussion

### 3.1. Embedding micro-obstacle structure to strength of secondary flow

First, we investigated the effects of obstacles on the flow field in curved channels because the strength and distribution of secondary flow significantly affect particle inertial focusing position and pattern [35,36, 44]. We numerically investigated the effects of micro-obstacles on the flow field in the main flow direction and within the channel cross-sections, Fig. 1(B) and (C). The obstacle structures can significantly boost the local main flow velocity by incorporating micro-obstacles within the channels to form local constrictions, Fig. 1 (B). The maximum velocity in the obstacle region (*r* = 75 μm) is almost 4 times that of the plain channel without any obstacles, Fig. 1(B). In addition, from the cross-sectional secondary flow fields before, within and after the obstacles (O-A, O–B, and O–C), we could observe that locally enlarged secondary flows are induced by the presence of obstacles, and the distribution of secondary flows is also altered, Fig. 1(C).

Furthermore, we designed four obstacle radii (*r*) of 0, 25, 50, and 75 μm and studied the influence of obstacle size on secondary flow in more detail. The average secondary flow velocities within three different cross sections (O-A, O–B, and O–C) all increase almost linearly with increasing obstacle size, Fig. 1(D). Besides, both the Y-axis and Z-axis components of the secondary flow are enhanced in direct proportion to the obstacle size, Fig. 1(E). As we know, the Y-axis component of secondary flow facilitates inertial focusing of particles toward the channel center, enabling the particle central focusing at a relatively lower flow rate. In contrast, the Z-axis component acts as a hindrance and may lead to particle defocusing due to its mixing effects [43,45]. Therefore, the obstacle size can adjust the strength of secondary flow and potentially affect the particle inertial focusing and separation in sinusoidal channels. In the following sections, we experimentally tested the effects of obstacles on particle inertial focusing behavior.

### 3.2. Advancing the central particle focusing by micro-obstacle structures

**Fig. 2.**
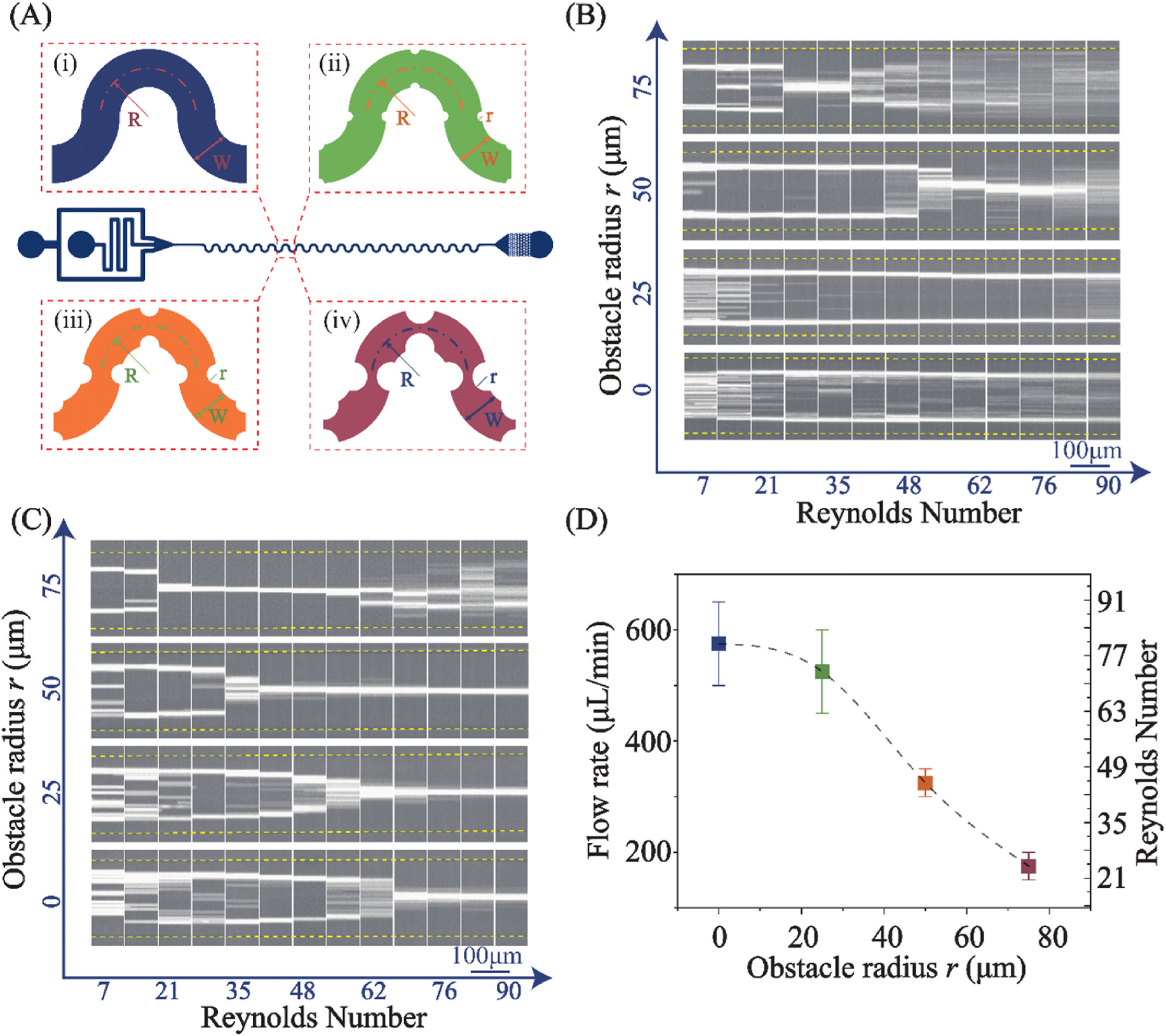
(A) Schematics of symmetric sinusoidal microchannels with (i) no obstacle, (ii-iv) with obstacles of 25, 50 and 75 μm in radius. (B) The distribution of 5-μm and (C) 10-μm polystyrene particles in sinusoidal channels with different obstacle sizes under different flow Reynolds numbers. (D) The working flow rate ranges for effective separation of 5-μm and 10-μm particles in channels with varying obstacle sizes.

We experimentally investigated the effect of micro-obstacle structures on particle inertial focusing behavior in sinusoidal channels. The sinusoidal channels with four different obstacle sizes of *r* = 0, 25, 50 and *r* = 75 μm were designed and fabricated, Fig. 2(A). 5-μm and 10-μm fluorescent spherical polystyrene beads were dispersed in DI water and were respectively infused into each channel with flow rate ranging from 50 to 650 μL/min with an incremental step of 50 μL/min. The focusing behaviors of 5 and 10 μm particles at different flow rates were plotted in Fig. 2(B) and (C), respectively. We observed that the particle focusing pattern in the sinusoidal channels with obstacles is similar to the plain sinusoidal channels without obstacles. Firstly, particles focused near both sidewalls of the channel under relatively low Reynolds number conditions. As Reynolds number increases, particles gradually migrate to the channel centre and form a single central focusing streak. If the Reynolds number exceeds certain thresholds, the particle trajectories gradually disperse again. Moreover, the larger the obstacle size was, the earlier the central focusing pattern occurred. This is because that obstacle structure can enhance the strength of Dean flow and lead to the advancement of central focusing at a lower flow rate. The strength of Dean flow increases with the increasing obstacle radius, as evidenced by the simulation results depicted in Fig. 1(D).

We also determined the working flow rate range for effective separation of 5-μm and 10-μm binary particle mixture in sinusoidal channels with different obstacle sizes, Fig. 2 (D). 10-μm particles migrate to the channel centre as a single focusing streak at a much lower flow rate than that for the 5 μm particles. Within a certain flow rate range, 5-μm particles still align near the two sidewalls so that the distinct positions at the outlet can separate 5-μm and 10-μm particles. Therefore, the working flow range is determined under which 10-μm particles already focus at the channel centre, while 5-μm particles align near two sidewalls. From Fig. 2(D), we can observe that the larger obstacle size results in a lower working flow rate to separate the same binary mixture solution. This superior property will be helpful in developing cascaded channels where the working flow rate in each stage needs to be adjusted to enable the proper functioning of channels in both upstream and downstream stages, as demonstrated in the following section.

### 3.3. Enhancing separation efficiency of 5-μm and 10-μm polystyrene particles via a cascaded platform

In this work, we developed a cascaded microfluidic device consisting of two sinusoidal channels to process samples twice continuously in a single runtime. Generally, having a high-purified outcome with only one-time processing is challenging, especially when vast background particles exist. The conventional way is to re-circulate the sample from the target outlet to purify the sample again [3]. However, manual operation to reload samples from outlets to inlets may cause sample loss and cross-contamination. Therefore, it is more promising to integrate two stages in one platform directly, achieving two processes in a continuous manner. Since fluid flow divides at the end of the first channels, and only a portion of fluid flows into the second stage downstream, the flow rate becomes much lower. Therefore, we incorporated micro-obstacle arrays in the secondary sinusoidal channels to adjust the working flow rates so that both stages can function properly.

**Fig. 3.**
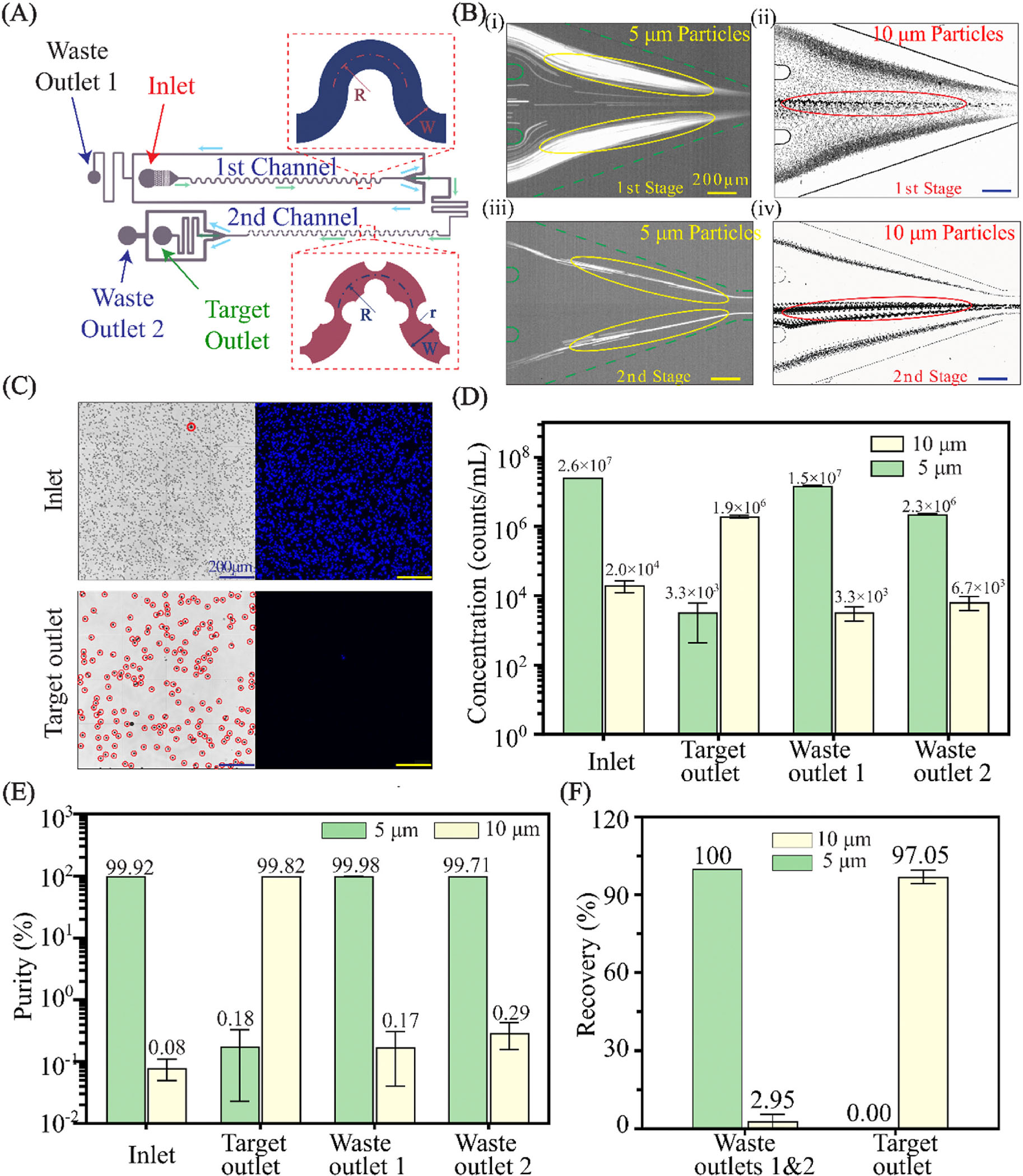
(A) The cascaded device consists of a plain sinusoidal channel as the first stage and a sinusoidal channel with concave obstacles (*r* = 75 μm) as the secondary stage. The arrow indicates the flow direction. (B) Particle trajectories at the bifurcation regions at the 1st and 2nd stages, respectively. The left-sided images show the fluorescent trajectories of 5 μm particles, and the right-sided brightfield images show the trajectories of both 5 and 10 μm particles. (C) Microscopic images of particle mixtures under a hemocytometry, before and after single processing via the cascaded chip. The blue fluorescence colour represents the 5 μm particles, and the 10 μm particles are circled in red. (D) The concentration and (E) purity of particles before and after a single processing through the cascaded chip. (F) The recovery of 5 and 10 μm particles after separation in the cascaded chip. The flow rate is 560 μL/min (Re = 77).

In the developed cascaded microfluidic device, the first stage is a plain sinusoidal channel, and the second stage is a sinusoidal channel with obstacles, Fig. 3(A). The inlet of the second-stage channel was connected to the middle outlet of the first-stage channel so that the samples processed and collected at the middle outlet could be further separated in the second stage. Specifically, in the first-stage plain channel, the effective separation range was from 500 to 650 μL/min (*Re* = 76–90), and the fluid division ratio was 1:2 (middle outlet: sided outlets). This allocated around one-third of the flow to the second-stage channel, resulting in an input flow rate of 160–210 μL/min (Re = 22–29). To facilitate the proper function of the second stage, obstacles with a radius of 75 μm were incorporated based on the working flow rate chart, Fig. 2(D).

Subsequently, we used a binary mixture of 5 and 10 μm to evaluate the separation performance of the cascaded chip, Fig. 3(B) and (C) and Video S1. To identify each particle trajectory, we used 5-μm fluorescent particles and 10-μm non-fluorescent particles. The ratio of 5-μm and 10-μm particles in the binary mixture is approximately 1000:1, representing the ratio between RBCs and WBCs in the whole blood [46]. The working flow rate was 560 μL/min (Re = 77), and the particle trajectories were captured by the high-speed camera under both fluorescent and bright fields, Fig. 3(B). In the first stage, most 5-μm particles were depleted from sided outlets, but still a small residual of 5-μm particles followed the trajectories of 10-μm particles and entered the middle outlet, Fig. 3B (i) and (ii). In the second stage, the further purification process happened, where residual 5-μm particles were completely removed, and the purity of 10-μm particles was significantly increased, Fig. 3B(iii) and (iv).

To quantively evaluate the separation performance, the separated particles from three outlets were collected and counted by a haemocytometer, Fig. 3(C)–(F). At the inlet, the 5-μm particles with blue-fluorescent color dominated, and it was hard to identify the 10-μm particles. After a single pass through the cascaded device, the device removed a massive amount of 5 μm particles, and the target outlet collected the majority of 10 μm particles. The purity of 10 μm particles increased from 0.08 % to 99.83 %, with a 1247-fold enrichment, Fig. 3 (E). Meantime, the recovery ratio of 10 μm particles was nearly 97.05 % for the cascaded chip, Fig. 3(F). This outstanding performance lays the foundation for the high-efficiency separation of blood cells for clinical applications.

### 3.4. High-purify WBC isolation from blood using a cascaded device

**Fig. 4.**
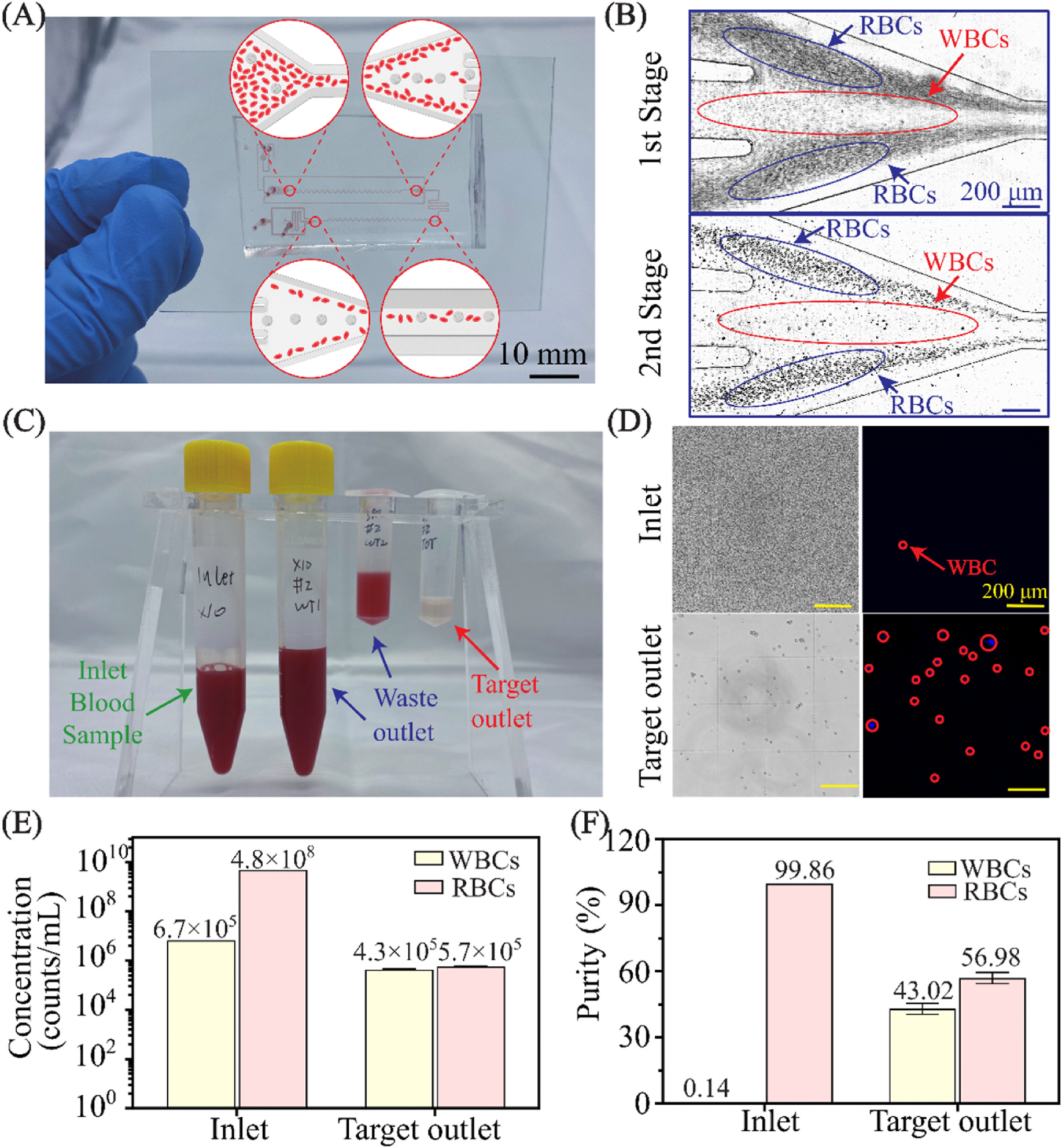
(A) A photo of the microfluidic device with cascaded sinusoidal channels. (B) The brightfield images of blood cells at the bifurcation regions of the 1st and 2nd stage, respectively. (C) The photos of collected blood samples from the inlet and three outlets. (D) Microscopic images of blood mixtures at the inlet and the target outlet after single processing via the device. The blue fluorescence colour represents the WBCs with DAPI staining. (E) The concentration and (F) purity of RBCs and WBCs before and after single processing through the device. The whole blood at the inlet is diluted 10 times. The infusion flow rate is 560 μL/min (Re = 77).

In a standard blood sample, RBCs occupy 40–45 % of total blood volume, while the volume of WBCs accounts for only 1 %. The quantity ratio of RBCs to WBCs is around 1000:1 [4]. The efficient removal of the massive background RBCs and the high purification and enrichment of WBCs are critical for many clinical applications. We applied our cascaded device to separate WBCs from the whole blood samples, Fig. 4 (A). We diluted the whole blood sample 10 times using HBSS solution and infused the diluted blood sample into our device at 560 μL/min (Re = 77). At the first purification stage, most of the dense RBCs and platelets were continuously depleted from the sided outlets while a small portion still escaped into the central outlet, Fig. 4(B) and Video S2. In the second stage, the total number of cells decreased significantly, and the RBCs were further purified and removed from the waste outlets.

Furthermore, we characterised the separation performance by collecting the blood samples before and after processing. The excellent separation performance is reflected in the colours observed in the collected blood samples within the tubes, Fig. 4(C). The collected blood samples were also observed under a hemocytometer, Fig. 4(D). We used the DAPI dye to stain WBCs exclusively to distinguish WBCs from RBCs. Since RBCs and platelets have no nucleus, only WBCs are stained with DAPI, shown in blue [47]. Extensive elimination of the background blood cells (RBCs and platelets) and significant enrichment of WBCs can be observed in Fig. 4(D). At the inlet, we can see a massive number of background cells, and it is challenging to identify WBCs due to the rarity of WBCs in whole blood. After only one processing, we can observe the WBCs clearly, and RBCs and platelets were removed remarkably. The background cells (mainly RBCs) have been significantly depleted from 4.8 × 10^8^ counts/mL to 5.7 × 10^5^ counts/mL, with almost 3 order RBCs depletion. Moreover, the purity of the WBCs was enhanced from 0.14 % to 43.017 %, with a 307-fold enrichment only after single processing, Fig. 4(E) and (F). The recovery of WBCs was 17.7 %.

We found that the separation performance on blood cells was not as good as that of polystyrene particles in Fig. 3. There are several possible reasons. First, cell concentration in blood is much higher than in bead mixture, and a much more intensive cell interaction may happen when cells are moving inside the microchannels. Second, RBCs are not spherical, and blood cells are much more deformable than rigid polystyrene beads. Moreover, WBCs are not uniform in size but range from 8 to 12 μm. All these factors affect cell migration and focusing and result in a lower separation purity and efficiency than spherical particles with uniform sizes. To further improve WBC recovery, we could recirculate the sample through the cascaded channel or connect additional units for multi-stage processing. However, a key challenge remains in effectively connecting different units in a cascaded configuration. With each added unit, the complexity of flow resistance control increases. For instance, the third stage requires a flow rate nearly one order of magnitude lower than the inlet flow rate, making efficient performance matching across all three units especially challenging. Alternatively, we could integrate external mechanisms such as dielectrophoresis (DEP), magnetophoresis (MP), or acoustic forces to optimize downstream stages, as these techniques operate effectively at lower flow rates and offer precise control over separation. However, these active methods complicate system design and add external components, increasing both complexity and cost.

## 4. Conclusion

In this work, we have developed a cascaded inertial microfluidic device with two sinusoidal microchannels in a series manner to isolate WBCs from the blood sample. We first quantified the effects of micro- obstacles on the flow field in the main flow direction and within the channel cross-section. By incorporating micro-obstacles within the channels to form local constrictions, the obstacle structures can significantly boost the local main flow velocity and enlarge the cross-sectional secondary flow. Next, we examined the working effect of obstacles on the particle focusing positions of 5-μm and 10-μm particles. We found that the size of micro-obstacles can tune the flow rate for particle inertial focusing and separation. Subsequently, we developed a cascaded device with two sinusoidal microchannels, keeping both channels functioning adequately simultaneously based on this benefit of obstacle structures. Then, we used a binary particle mixture (5 μm and 10 μm) with a ratio of around 1000:1 to investigate the separation performance of the device with cascaded channels. After single processing through the device, the purity of 10-μm particles increased from 0.08 % to 99.83 %, with a 1247-fold enrichment in 560 μL/min flow rate (Re = 77). In addition, we tested the separation performance using diluted (× 1/10) whole blood. The purity of the WBCs was enhanced from 0.14 % to 43.017 %, with a 307-fold enrichment in a single process. Meanwhile, the background RBCs have been significantly removed from 4.8 × 10^8^ to 5.7 × 10^5^ counts/mL, with ~3 orders of depletion rate. We believe that the cascaded-style device has the potential to provide high purity and high efficiency of blood cells separation for clinical diagnosis and therapeutics.

## Supplementary Material

Supporting information High-efficient white blood cell separation from whole blood using cascaded inertial microfluidics

Video 1

Video 2

## Data Availability

Data will be made available on request.

## Appendix A. Supplementary data

Supplementary data to this article can be found online at https://doi.org/10.1016/j.talanta.2024.127200.
