# Supplementary material for: High-efficient white blood cell separation from whole blood using cascaded inertial microfluidics
Source: Talanta. Author manuscript; Available in PMC 2026 Jul 11. (PMC13355906; doi:10.1016/j.talanta.2024.127200)
Supplement: Supporting information High-efficient white blood cell separation from whole blood using cascaded inertial microfluidics [file NIHMS2182446-supplement-Supporting_information___High-efficient_white_blood_cell_separation_from_whole_blood_using_cascaded_inertial_microfluidics.docx]

**Supporting information**

**High-efficient white blood cell separation from whole blood using cascaded inertial microfluidics**

Haotian Cha^+*a^, Xiaoyue Kang^+b^, Dan Yuan^b^, Belinda de Villiers^c^, Johnson Mak^c^, Nam-Trung Nguyen^*a^, Jun Zhang^*a,d^

^a^ Queensland Micro- and Nanotechnology Centre, Griffith University, Nathan, Queensland 4111, Australia. E-mail: [h.cha@griffith.edu.au](mailto:h.cha@griffith.edu.au); [jun.zhang@griffith.edu.au](mailto:jun.zhang@griffith.edu.au); [nam-trung.nguyen@griffith.edu.au](mailto:nam-trung.nguyen@griffith.edu.au)

^b^ School of Mechanical and Mining Engineering, The University of Queensland, Brisbane, QLD 4072, Australia

^c^ Institute for Glycomics, Griffith University, Gold Coast, Queensland, Australia

d. School of Engineering and Built Environment, Griffith University, Nathan, Queensland 4111, Australia

⁺ Equally contributed.

**Table of Contents**

**Supplementary video S1**: Trajectories of 5 and 10 μm at the bifurcation region of cascaded sinusoidal channels

**Supplementary video S2:** Trajectories of white blood cells (WBCs) and bloods cells at the bifurcation region of cascaded sinusoidal channels
